# Ni-Rich Layered Oxide with Preferred Orientation (110) Plane as a Stable Cathode Material for High-Energy Lithium-Ion Batteries

**DOI:** 10.3390/nano10122495

**Published:** 2020-12-11

**Authors:** Fangkun Li, Zhengbo Liu, Jiadong Shen, Xijun Xu, Liyan Zeng, Yu Li, Dechao Zhang, Shiyong Zuo, Jun Liu

**Affiliations:** Guangdong Provincial Key Laboratory of Advanced Energy Storage Materials, School of Materials Science and Engineering, South China University of Technology, Guangzhou 510641, China; mslifk@scut.edu.cn (F.L.); 201810103813@mail.scut.edu.cn (Z.L.); 201910103734@mail.scut.edu.cn (J.S.); xuxijun2019@scut.edu.cn (X.X.); 201820117753@mail.scut.edu.cn (L.Z.); 201820117766@mail.scut.edu.cn (Y.L.); 201810103808@mail.scut.edu.cn (D.Z.); shyzuo@scut.edu.cn (S.Z.)

**Keywords:** Li-ion batteries, Ni-rich layered cathode, preferred orientation, diffusion dynamics, voltage drop

## Abstract

The cathode, a crucial constituent part of Li-ion batteries, determines the output voltage and integral energy density of batteries to a great extent. Among them, Ni-rich LiNi_x_Co_y_Mn_z_O_2_ (x + y + z = 1, x ≥ 0.6) layered transition metal oxides possess a higher capacity and lower cost as compared to LiCoO_2_, which have stimulated widespread interests. However, the wide application of Ni-rich cathodes is seriously hampered by their poor diffusion dynamics and severe voltage drops. To moderate these problems, a nanobrick Ni-rich layered LiNi_0.6_Co_0.2_Mn_0.2_O_2_ cathode with a preferred orientation (110) facet was designed and successfully synthesized via a modified co-precipitation route. The galvanostatic intermittent titration technique (GITT) and electrochemical impedance spectroscopy (EIS) analysis of LiNi_0.6_Co_0.2_Mn_0.2_O_2_ reveal its superior kinetic performance endowing outstanding rate performance and long-term cycle stability, especially the voltage drop being as small as 67.7 mV at a current density of 0.5 C for 200 cycles. Due to its unique architecture, dramatically shortened ion/electron diffusion distance, and more unimpeded Li-ion transmission pathways, the current nanostructured LiNi_0.6_Co_0.2_Mn_0.2_O_2_ cathode enhances the Li-ion diffusion dynamics and suppresses the voltage drop, thus resulting in superior electrochemical performance.

## 1. Introduction

The pursuit of environmental protection and low carbon emission has been causing a daily increasing requirement of high-value ratio energy storage devices. Lithium-ion batteries (LIBs) with long cycle life, and high energy density and working potential have been occupying a high proportion of the commercial battery market [1,2,3]. Nevertheless, there is still a lack of appropriate cathode materials with ultra-stable cycle life and fast charge/discharge rates to meet the demand of next-generation batteries [4,5]. It is well known that the cathode considerably determines the whole energy density of batteries. Recently, Ni-rich LiNi_x_Co_y_Mn_z_O_2_ (x + y + z = 1, x ≥ 0.6) layered transition metal oxides have been emerging as the most potential cathode candidates for LIBs due to their relatively higher capacity and output working voltage than LiFePO_4_, more abundant resources, and lower cost than traditional LiCoO_2_ [6,7,8,9,10]. Unfortunately, its practical application still suffers from low cycle stability, severe voltage drops and poor kinetics [11,12]. There are many factors that including cation mixing, phase transition, loss of lattice oxygen, particle cracking, electrolyte decomposition, electrode/electrolyte parasitic reaction, transition metal dissolution and surface reconstruction could influence the voltage drops, charge transfer rate, and cycle-stability of cathode during long-term charge/discharge process [4,8,13,14,15,16,17,18]. To address these problems and promote its further development, various strategies have been proposed and evaluated, such as surface modification (including Li_3_PO_4_, ZnO, MgO, AlF_3_, Al_2_O_3_, TiO_2_, and ZrO_2_, etc.) [15,19,20,21,22,23], cationic (or anionic) doping (including Zr^4+^, Ti^4+^, Al^3+^, Mg^2+^, F^–^, and B^3+^, etc.) [21,22,24,25,26], concentration gradient and core-shell structure designing [27,28,29], and morphology control [30].

Among the current used multiple modification methods, nanocrystallization has been identified as the most effective measure to improve the kinetic property of electrode materials and is widely studied and adopted to enhance the electrochemical performance [27,31,32,33,34,35,36]. The advantages of nanostructured materials that offer short Li^+^ transport pathways and provide a large contact area for charge transfer. On the other hand, Ni-rich layered oxides possess a typical hexagonal α-NaFeO_2_ structure which offers two-dimensional (2D) channels for Li-ion migration along with the a (or b) axis during the charge/discharge process. Therefore, a large number of researchers are committed to improving electrochemical performance by exposing the active electrochemical facets [37,38,39]. Some crystal planes (i.e., (010), (100), and (110) planes) can provide an open structure for Li-ion diffusion and charge transfer while others do not. Certainly, a large number of studies have been published to support this point. Wu and co-workers verified that it was beneficial to enhance the electrochemical performance by synthesizing fusiform hierarchical particles with exposing (110) plane [40]. Moreover, the work of Notten’s research examined that it was beneficial to enhance the rate performance by exposing the more active {010} facets, which could afford the fast Li^+^ ion transmission rate [41]. Take all the above factors into account, synthesizing nanostructured materials with preferential orientation crystal planes will dramatically improve the poor kinetics, moderate the voltage drops and achieve superior charge/discharge performance.

Herein, Ni-rich LiNi_0.6_Co_0.2_Mn_0.2_O_2_ nanobricks cathode with a preferred orientation (110) electrochemical plane was synthesized via solid-state reaction by mixing Li source with Ni-Co-Mn composite oxide nanosheets. The nanosheets structure of Ni-Co-Mn composite oxide is originated from thermal decomposition of navel hydroxide precursor. The precursor with nanosheet structure is massively prepared by a modified co-precipitation route. This unique nanobrick morphology with more exposed (110) electroactive planes can offer an open structure for Li-ions diffusion. Additionally, the synergistic effect of the nano-size effect and the exposed (110) crystal plane significantly improve the electrochemical performance and endow superior dynamics. Especially, the voltage drop is as small as 67.7 mV in the current density of 0.5 C for 200 cycles. Hence, this rational strategy to improve performance is instructive for other materials.

## 2. Materials and Methods

### 2.1. Preparation of LiNi_0.6_Co_0.2_Mn_0.2_O_2_ (NCM622) Cathode Materials

Firstly, the hydroxide precursor nanosheets were synthesized via a modified co-precipitation route. 100 mL 20 mM feed solution prepared by mixing NiSO_4_·6H_2_O, CoSO_4_·7H_2_O, and MnSO_4_·H_2_O with the proportion of Ni: Co: Mn at 3:1:1. Then, the feed solution was dripped into a 50 mL NH_3_·H_2_O (pH = 11) solution three-necked flask with continuously stirring. Synchronously, 100 mL 30 mM Na_2_CO_3_ solution and 100 mL 0.6 mM NH_3_·H_2_O solution were completely put into the flask. After that, the temperature was maintained at 55 °C and stirring velocity controlled at 800 rpm for 24 h to accomplish the co-precipitation reaction procedure. Then, the precursors were filtered, washed three cycles alternately with ultrapure water and ethanol, and dried at 80 °C overnight. The oxide composite intermediates were obtained by pre-sintering hydroxide precursor at 600 °C for 5 h in a furnace. Finally, the mixture of as-synthesized oxide precursors and Li_2_CO_3_ with an appropriate proportion was calcined at 850 °C for 12 h in the air to obtain pristine LiNi_0.6_Co_0.2_Mn_0.2_O_2_, denoted as NCM622.

### 2.2. Characterization Methods 

The crystalline structure of these materials was determined by using a PANalytical Empyrean X-ray diffractometer (XRD, PANalytical B.V., Almelo, The Netherlands) equipment with Cu-K radiation (λ = 1.54056 Å, operating at 40 kV, 45 mA). The intensity data collected by XRD was analyzed by the Rietveld improved program-General Structural Analysis System-Ι (GSAS-Ι) software package. The morphology, microstructure and elemental distribution were measured by a field emission scanning electron microscope (SEM, Zeiss Gemini DSM 982, Carl Zeiss AG, LEO Oberkochen, Germany) equipped with an EDS energy dispersive X-ray spectrometer with an acceleration voltage of 15 kV. High-resolution transmission electron microscopy (HRTEM) data was obtained by a JEM-2100F (JEOL Co., Akishima City, Tokyo, Japan) instrument operating at 200 kV. N_2_ desorption and adsorption isotherms were evaluated at 77 K with a Quadrachrome adsorption instrument.

### 2.3. Electrochemical Measurements

The electrochemical properties were tested in CR2016 coin-type cells. These cells were assembled with the NCM622 cathode, Li metal anode, organic electrolyte, and polypropylene separators in an Ar-filled glove box. The organic electrolyte was that 1.0 M LiPF_6_ dissolved in ethylene carbonate/diethylene carbonate/dimethyl carbonate (EC/DEC/DMC, 1:1:1 in volume). The NCM622 electrode was prepared by thoroughly mixing the active material, Super-P, and polyvinylidene fluoride (PVDF) with a weight ratio of 8:1:1 in *N*-methyl(pyrrolidinone) (NMP). Then the slurry spread onto the aluminum foil and dried in a vacuum oven at 100 °C for 12 h. The mass loading of cathode materials was measured in the range of 1.6–2.2 mg cm^−2^. LAND CT2001A testing system (LAND Electronic Co. Ltd., Wuhan, China) was performed to evaluate the cycling performance and rate capacity at various current density in the voltage region of 2.8–4.4 V. Electrochemical impedance spectroscopy (EIS) was measured by an electrochemical workstation (Gamry Interface 1000, Gamry Electrochem. Instru. Co., Warminster, PA, USA) with an amplitude of 10 mV from 10^5^ Hz to 10^−2^ Hz. Galvanostatic Intermittent Titration Technique (GITT) was performed at a constant current pulse of 0.1 C rate for 15 min and then rest for 90 min to stabilize the cell voltage between 2.8 and 4.4 V.

## 3. Results and Discussion

**Figure 1:**
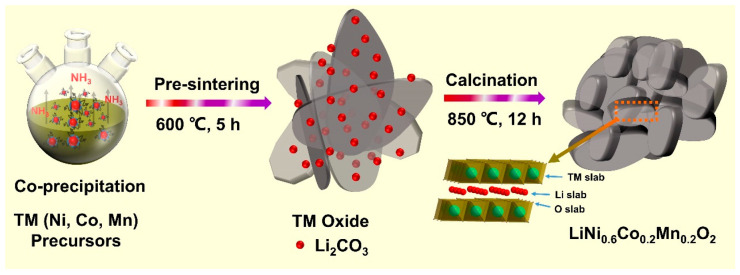
Schematic illustration of the synthesis process of nannobricks LiNi_0.6_Co_0.2_Mn_0.2_O_2_ (NMC622) cathode materials.

**Figure 2:**
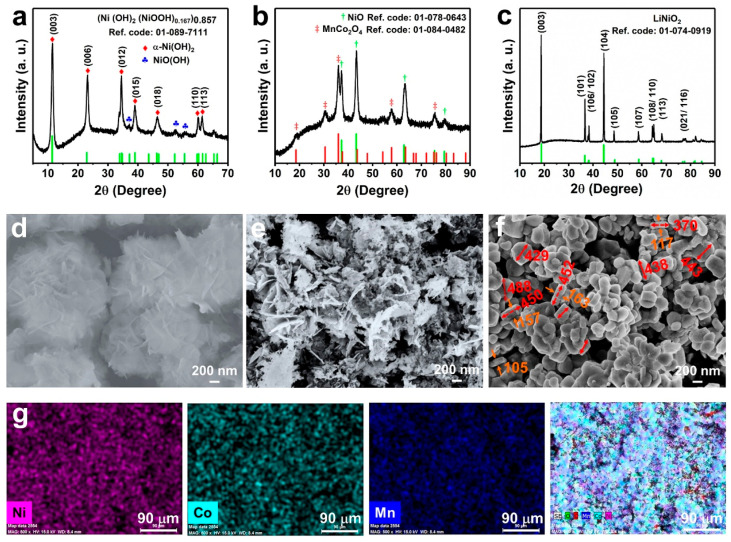
(**a**–**c**) XRD patterns of (**a**) (oxy)hydroxide precursor, (**b**) intermediate oxide, and (**c**) as-prepared NCM622; (**d**–**f**) Corresponding SEM images of (**d**) (oxy)hydroxide precursor, (**e**) intermediate oxide, and (**f**) NCM622 materials; (**g**) elemental EDS mapping of Ni, Co, Mn, and all elements of NCM622.

Figure 1 presents the detailed formation procedure of TM (Ni, Co, Mn) (oxy)hydroxide precursor and nanobricks LiNi_0.6_Co_0.2_Mn_0.2_O_2_ (marked as NCM622). First, The Mn/Co ions co-substituted Ni(OH)_2_ with nanoplates morphology was prepared by a modified co-precipitation route using a high-pH values ammonium hydroxide as the base solution. Subsequently, these (Ni, Co, Mn) hydroxide nanoplates were adopted as novel phase precursors for the formation of the final high nickel NCM622. XRD pattern (Figure 2a) verifies that this TM (Ni, Co, Mn) hydroxide can be precisely indexed to hexagonal α-Ni(OH)_2_ (JCPDS no. 38-0715) phase. According to the XRD results, there are two fundamental phases of Ni(OH)_2_ that exist in the precursor. It is interesting to note that the c-parameter of α-Ni(OH)_2_ is greater than that of β-Ni(OH)_2_ [42], which means that the diffraction angle of α-Ni(OH)_2_ is equivalent to a low angle shift of β-Ni(OH)_2_. This phenomenon is originated from the type of anions (OH^−^, CO_3_^2−^, SO_4_^2−^) and H_2_O molecules embedded in the Ni(OH)_2_ lattice [43,44,45,46]. Separately, the corresponding XRD parameters were listed in Tables S1−S3. The diffraction peaks of NiOOH were associated with the easy oxidization of α-Ni(OH)_2_ to NiOOH [47]. Therefore, the as-synthesized precursor is (oxy)hydroxide (TM(OH)_2_/TMOOH) and can be precisely indexed to (Ni(OH)_2_(NiOOH)_0.167_)_0.857_ (JCPDS no. 89-7111). As depicted in Figure 2d, the obtained (oxy)hydroxide precursor shows a hierarchical architecture, which is consists of randomly assembled nanosheets. To avoid destructive morphology change resulting from the crystal growth during solid-state lithiation and attain intermediate oxides composite, the (oxy)hydroxide precursor was pre-calcined at 600 °C in air condition [48]. The XRD result (Figure 2b) directly reveals the changes of (oxy)hydroxide to precursor NiO (JCPDS no. 78-0643) and MnCo_2_O_4_ (JCPDS no. 84-0482). As it appears in the SEM signals (Figure 2e), this oxide composite well inherited the nanosheets’ morphology. To gain an in-depth realizing of the intermediate’s elemental composition, SEM-EDS mapping was also performed. The EDS mapping signals reveal the uniform distribution and co-exists of Ni, Co, Mn, and O elements (Figure S1). Furthermore, and the atomic ratio of Ni, Mn, and Co is determined at approximately 3:1:1, which is well consistent with the designed stoichiometric values (Figure S2).

The structure and morphology of NCM622 materials after lithiation reaction were also investigated by XRD and SEM. Evidently, all the XRD diffraction peaks of NCM622 (Figure 2c) could be indexed to the layered hexagonal α-NaFeO_2_ single-phase with a space group of R$\bar{3}$m [49]. On account of previous studies, the splitting of (006)/(102) and (108)/(110) peaks indicates this NCM622 cathode with a well-ordered layered structure [33,50]. Certainly, no distinct extra peaks appear in the XRD patterns suggesting the attained NCM622 cathode without any impurity phase. Figure 2f displays the SEM result of NCM622 products, which confirm that the as-prepared cathode with an architecture nanobrick morphology possesses smooth surfaces and enhanced sidewalls. Separately, the thickness of these nanobricks can be intuitively acquired as about 300 nm. Additonally, EDS mapping signals of Ni, Co, Mn, and all elements (Figure 2g) verify that all elements are uniformly distributed. It is worth noting that the thickness difference between the NCM622 and precursor nanosheet is associated with the merging of the multilayer boards during the high-temperature reaction [41]. The unique hierarchical structure is not only effectively forms good penetration of electrolytes, but also markedly increases the transport pathway for Li-ion diffusion during the delithiation/lithiation processes. To accurately verify the element composition of Li:Ni:Co:Mn in the nanobricks, ICP-OES (Agilent 720ES) measurements were performed and provided in Table S4. The results demonstrate that the molar ratio of Li:Ni:Co:Mn in nanobricks is well consistent with the expected stoichiometric ratio of LiNi_0.6_Co_0.2_Mn_0.2_O_2_.

**Figure 3:**
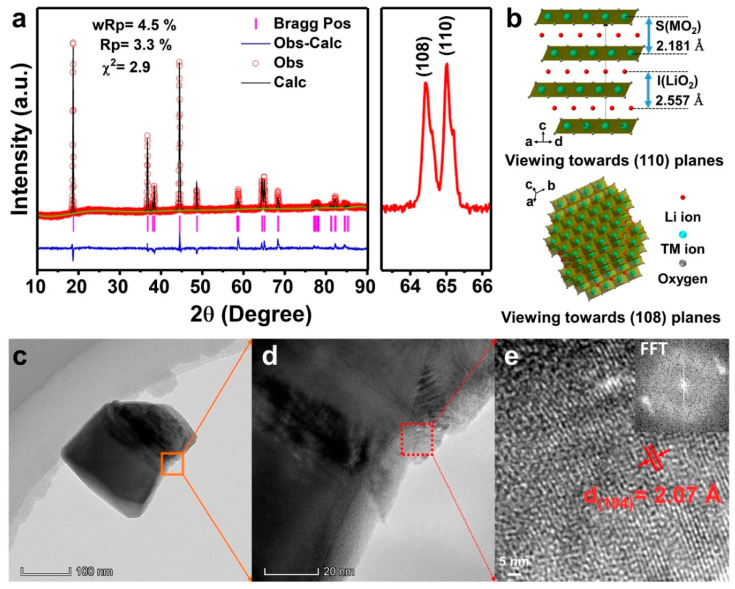
(**a**) Refined XRD pattern of NCM622 material based on LiNiO_2_ hexagonal (R$\bar{3}$m) phase and magnified view of (108) and (110) peaks; (**b**) The atomic structure of NCM622 vertical viewed (110) and (108) planes; (**c**–**e**) TEM, HRTEM images and FFT pattern (inset) of NCM622. Note: Z_ox._ is the position of O^2−^ along the c axis in a hexagonal unit cell. Typically, (0, 0, Z_ox._) is used as the coordinate for O^2−^. The slab thickness S(MO_2_) and the interslab thickness I(LiO_2_) correspond to the distances along the c_hex_ axis between the oxygen layers of the (Ni, Co, Mn)O_2_ slab and LiO_2_ interslab spaces, respectively. They are defined as S(MO_2_) = (2/3 − 2Z_ox._)c_hex_. and I(LiO_2_) = (c_hex_/3) − S(MO_2_) [54].

To gain more insight into the crystal structure, the XRD pattern of this NCM622 cathode was refined and analyzed by using GSAS software. Figure 3a and Table S5 display the refined results of NCM622 material based on hexagonal phase α-NaFeO_2_ (R$\bar{3}$m) layered structure. It is worth noting that the intensity of (110) peak is stronger than (108) peak in the magnified view, and the ratio values of I_(110)_/I_(108)_ is 1.06. The ratio of I_(110)_/I_(108)_ over 1.0, which further indicates the abundance of exposed electroactive (110) facets NCM622 cathode possessed. This phenomenon has been reported and verified in many previous works, in which the preferential growth of the crystal structure with an exposed plane is believed to enhance the electrochemical performance [39,40,51,52,53]. This particular structure with more exposed (110) facets of NCM622 cathode is believed to offer unobstructed Li^+^ diffusion channels. The microstructure of NCM622 was characterized by transmission electron microscopy (TEM), high-resolution TEM (HRTEM) and fast Fourier transform (FFT). Figure 3c further verifies that the resulted NCM622 with a nanobrick structure, which is well inherited the structure from the precursor. The HRTEM image and inset FFT pattern (Figure 3e) with an interplanar distance of 2.07 Å is assigned to the (104) planes of NCM622 cathode materials.

**Figure 4:**
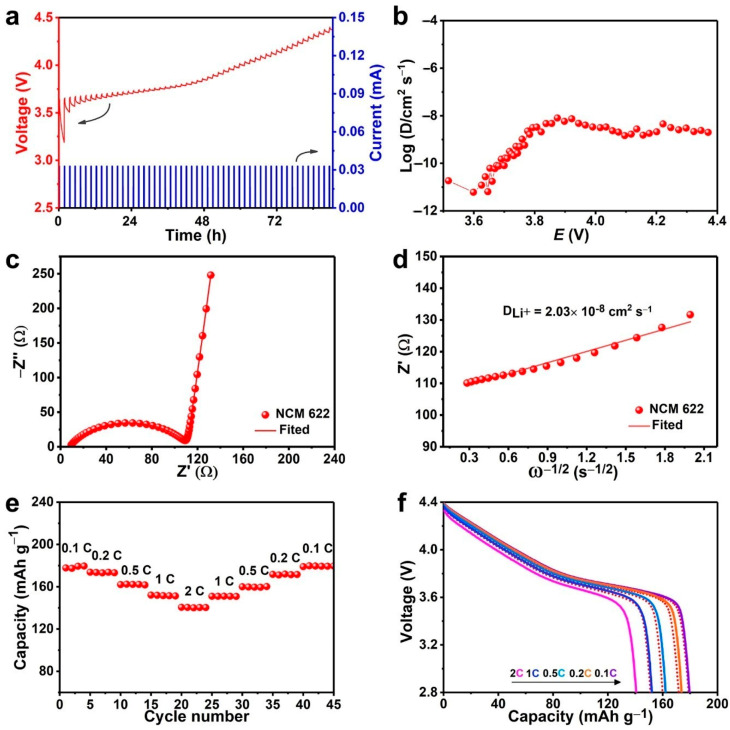
(**a**) The GITT curves of NCM622 cathode in the voltage of 2.8–4.4 V at C/10 rate and (**b**) corresponding variations in Li^+^ diffusion coefficient as functions of the potential during the charging processes; (**c**) Nyquist plots; (**d**) the relationship between Z_re_ and ω^−1/2^ at low frequencies; (**e**) rate capability of NCM622 electrode at different discharge rates (0.1 C, 0.2 C, 0.5 C, 1 C, 2 C, 1 C, 0.5 C, 0.2 C, 0.1 C) in the voltage of 2.8–4.4 V and (**f**) corresponding discharge voltage profiles at various rates.

The intrinsic lithium-ion diffusion dynamic properties of current NCM622 with preferred orientation (110) facets structure was further characterized. Galvanostatic intermittent titration technique (GITT) and electrochemical impedance spectra (EIS) were carried out to analyze the Li-ion diffusion coefficient, which is the key indicator for ion transport kinetics. The D_Li_^+^ is calculated by Equation (1) [30,55,56]:

(1) $$D_{Li^{+}}=\;\frac{4}{\pi \tau }{\left(\frac{n_{M}V_{M}}{S}\right)}^{2}{\left(\frac{\Delta E_{s}}{\Delta E_{t}}\right)}^{2}\left(\tau \leq \frac{L^{2}}{D_{Li^{+}}}\right)$$

where $D_{Li^{+}}$ is the Li^+^ diffusivity (cm^2^ s^−1^), *τ* is the time duration of the pulse (s), *n*_M_ and *V*_M_ are the molar mass (mol) and volume (cm^3^ mol^−1^) of the active material, *S* is the cell interfacial area (cm^2^), respectively. Δ*E*_s_ is the potential difference at the state of equilibrium (V), and Δ*E_t_* is the polarization potential (V), and *L* is the length of Li^+^ diffusion (cm). The applied current plus vs. cell voltage for a single titration step of GITT curves are extracted out in Figure S3, in which the different parameters of V_o_, IR _drop_, V_1_, V_2_, V_3_, etc., are schematically marked out. The GITT values in the entire interval charge state are shown in Figure 4a and the corresponding calculated D_Li_^+^ of NCM622 electrode lies in the range of 10^−12^–10^−8^ cm s^−1^ (Figure 4b).

Furthermore, the EIS tests were also measured and depicted in Figure 4c. The D_Li_^+^ is further evaluated from the Warburg impedance date according to the following Equation (2) [57]:

(2) $$D_{Li^{+}}=\frac{R^{2}T^{2}}{2A^{2}n^{4}F^{4}C^{2}\sigma^{2}}$$

Z’= R_D_+ R_L_+ σω^−1/2^ (3)

where *R* is the ideal gas constant, *T* is the absolute temperature, *F* is the Faraday constant, n is the number of electrons per molecule oxidized, *C* is the concentration of Li^+^ in the cathode. *A* is the surface area of the electrode, which was determined by Brunauer-Emmett-Teller (BET) measurement using Quadrachrome adsorption isotherms at 77 k. As shown in Figure S4, the value of A was concluded as 5.92 m^2^ g^−1^. σ is the Warburg coefficient related to Z’ in Equation (3), which is the fitted slope of the relationship between the Z’ and the square root of frequency (ω^−1/2^) (Figure 4d). On account of Equations (2) and (3), D_Li+_ of NCM622 cathode could be calculated as 2.03 × 10^−8^ cm^2^ s^−1^. This value is also in good agreement with the results obtained by GITT. It is illustrated that the materials with preferred orientation (110) active facets have favorable Li-ion diffusion kinetics, which strongly supports the excellent electrochemical performance NCM622 cathode achieved.

To validate the rate capability and cycling performance NCM622 reached, the NCM622//Li half-cells were measured. Figure 4e,f show the rate capability of NCM622 at various charge-rates in the voltage range of 2.8–4.4 V. This NCM622 cathode retains a capacity of 178.6, 173.8, 162.1, 152.1, 140.6, 1551.1, 160.0, 171.7, and 179.0 mAh g^−1^ at 0.1, 0.2, 0.5, 1, 2, 1, 0.5, 0.2, and 0.1 C, respectively. When the current density returns to 0.1 C (from 1 to 0.1 C), yielding a capacity retention of 99%. The distinguished capacity retention proves that this NCM622 material possesses excellent rate capability. The superior performance also is an echo with previous GITT and EIS analysis. These superior properties can be attributed to the unique nanobrick structure with more exposed electroactive (110) facets and thereby reduced the diffusion distance, thus improving the Li^+^ diffusion kinetics. 

**Figure 5:**
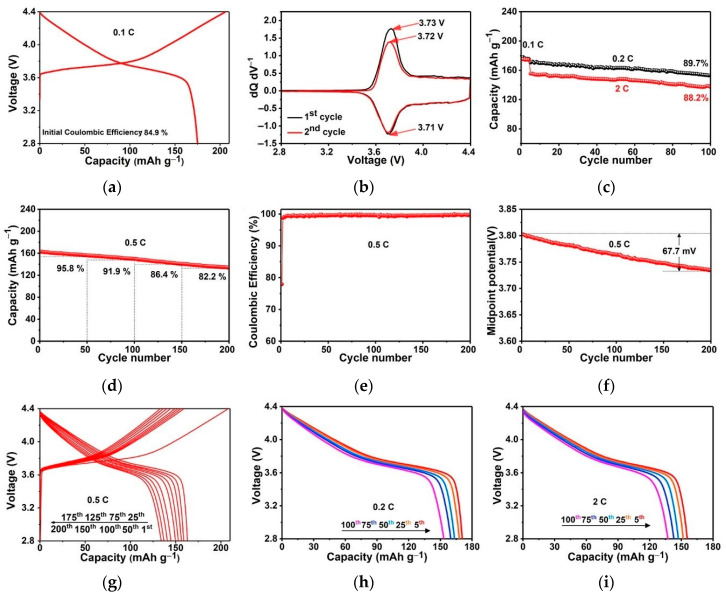
Electrochemical performance of NCM622 cells at 30 °C in the region of 2.8–4.4 V: (**a**) Typical charge/discharge curves for the first cycle at 0.1 C rate; (**b**) Differential capacity curves of the first and second charge/discharge cycles; (**c**) cycling performances at 0.2 C and 2 C after four activation cycles at 0.1 C and (**h**,**i**) their corresponding discharge voltage profiles; (**d**) long-term cycling performance at 0.5 C for 200 cycles and (**e**) corresponding coulombic efficiencies; (**f**) midpoint potential; and (**g**) charge/discharge voltage profiles.

The cycling stability of NCM622 nanobriks cathode was also evaluated and displayed in Figure 5. Figure 5a shows the smooth charge/discharge curves of NCM622 for the first cycle. It delivers a high initial discharge capacity of ~175 mAh g^−1^ and coupled with an initial coulomb efficiency (ICE) of 85% at a current density of 0.1 C. Moreover, at a certain high discharge voltage, this NCM622 cathode has a negligible discharge open circuit voltage drop of only 8.3 mV. To further unveil the charge/discharge mechanism, the corresponding differential capacity (dQ dV^−1^) curves as a function of cell voltage is provided in Figure 5b. The differential capacity curves mainly consist of a couple of redox peaks and display lower anodic peak voltages in the second charging process. Besides, the prepared NCM622 possesses a small voltage interval between the anodic and corresponding cathodic peaks indicating almost negligible polarization and well reversibility of this cathode [14]. Gratifyingly, the NCM622 material displays stable cycling performance (Figure 5c) and achieves superior capacity retention of 89.7% and 88.2% after 100 cycles at 0.2 C and 2 C, respectively. The corresponding discharge curves displayed in Figure 5h,i which vividly appear as stable discharge platforms and capacities without obvious drops and decay. As shown in Figure 5d–g, the long-term cycle stability of NCM622 was also measured at 0.5 C. Figure 5d exhibits a detailed analysis of the capacity retention of NCM cathode during the whole 200 cycles, which is conducted out through the recorded number of cycles (50 times interval) displaying a capacity retention as high as 82.2% after 200 cycles. The ICE of the nickel-rich layered cathode materials was associated with the side reactions and irreversible phase transitions at the electrode/electrolyte interface during the delithiation process. Especially for nano-sized particles, which possess highly active electrode/electrolyte interface thereby the side reactions are inevitable take place, thus causing part of the capacity irreversibly [11,15,16]. The coulombic efficiencies are all close to 100% in subsequent cycles, revealing that the NCM622 undergoes a highly reversible electrochemical reaction during the whole cycling process (Figure 5e). Figure 5f exhibits a small midpoint potential difference of 67.7 mV, suggesting a low voltage drop, thus delivering excellent long-term cycle stability. The corresponding charge/discharge curves (Figure 5g) directly present the voltage platform change reflecting the high-capacity retention this NCM622 achieved. The high-capacity retention and low voltage drop can be ascribed to the abundance of exposed (110) planes, which offers an open structure for rapid Li-ions transportation and ensures a small polarization.

Also, the structure and morphology of the electrode after cycling were investigated by XRD and SEM measurements. The XRD pattern of the NCM622 electrode (Figure S5(a)) in the voltage range of 2.8–4.4 V at 0.5 C rate after 200 cycles reveals that the NCM622 electrode still preserve a hexagonal crystal structure relating to the R$\bar{3}$m space group. Furthermore, the corresponding SEM images (Figure S5(b)) indicate NCM622 cathode still maintained the nanobrick morphology with a smooth surface and sharp edges. The XRD pattern and SEM images evidence that the rational structure design well protects the structural integrity of NCM622 from destruction.

**Figure 6:**
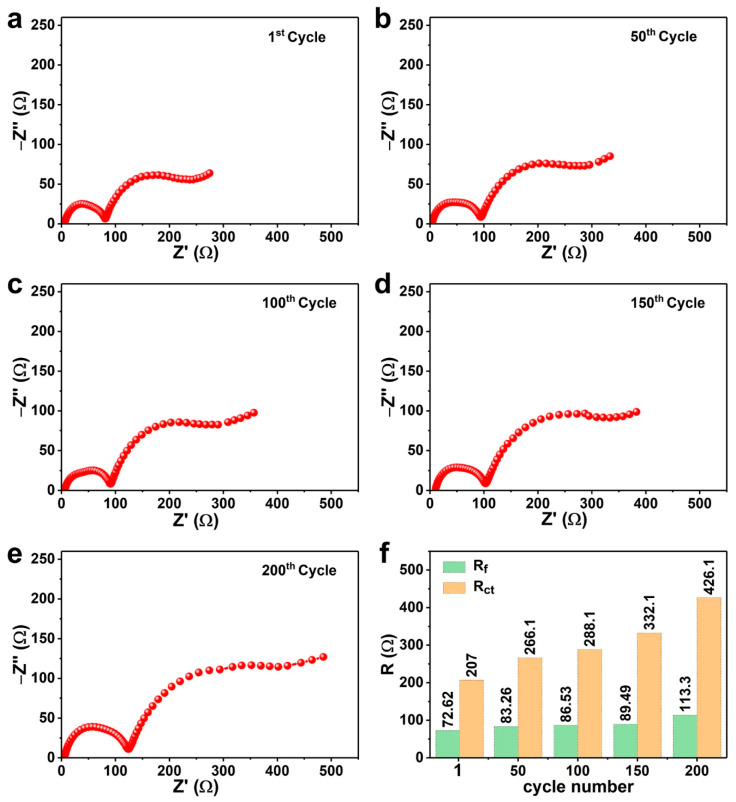
Nyquist plots and fitting curves of the NCM622 electrode after (**a**) 1st, (**b**) 50th, (**c**) 100th, (**d**) 150th, and (**e**) 200th cycles at 0.5 C rate in the voltage range of 2.8–4.4 V, and (**f**) Corresponding R_f_ and R_ct_ values were fitted by the equivalent circuit.

To acquire more helpful information revealing the possible changes NCM622 undergoes, the AC impedance measurements were evaluated at various cycles at a 0.5 C rate. As demonstrated in Figure 6a–e, the semicircles of high and medium frequency features can be observed for all Nyquist plots. The high and medium frequency region are associated with R_f_ and R_ct_, respectively. Based on the equivalent circuit (Figure S6), The R_f_ and R_ct_ values can be calculated and offered in Figure 6f. It can be observed that the R_f_ values without significant changes from 50th to 150th cycles suggest that the current cathode possesses stable cathode electrolyte interface (CEI) film during the cycle process. The R_ct_ value with continuous increase throughout the cycle is caused by the oxidative decomposition of the electrolyte on the electrode surface.

## 4. Conclusions 

Ni-rich NCM622 nanobricks with preferred orientation (110) active planes are successfully synthesized via a scalable approach, which consists of a modified co-precipitation procedure followed by a solid lithiation reaction. This NCM cathode achieves superior rate capability retaining a discharge capacity of 178.6, 173.8, 162.1, 152.1, and 140.6 mAh g^−1^ at 0.1, 0.2, 0.5, 1, and 2 C, respectively. The GITT and EIS analysis reveal that this NCM622 exhibits a good kinetic property equipped with a Li-ion diffusion coefficient around 2.03 × 10^−8^ cm^2^ s^−1^ and a sufficiently small voltage drop (67.7 mV) at 0.5 C after 200 cycles, and superior capacity retention, ca. 89.7% (0.2 C) and 88.2% (2 C). The superior Li^+^ storage performance NCM622 possessed could be attributed to the unique architectures with a preferred orientation (110) facet, which not only endows more channels for ion/electros transporting, but also reduces the diffusion distance and, thus, resulting in excellent diffusion dynamics and attaining superior electrochemical performance. This imaginative design sheds a light on constructing multistage structured cathode materials for the next-generation batteries.

## Supplementary Materials

The following are available online at https://www.mdpi.com/2079-4991/10/12/2495/s1, Figure S1: SEM image and elemental EDS mapping of Ni, Co, Mn, O and all elements for the intermediate oxides composite, the scale bar is 90 μm in all figures, Figure S2: EDS spectrum and corresponding element composition of intermediate oxides composite, Figure S3: Applied current plus vs. cell voltage for a single titration step of GITT curves, Figure S4: N_2_ adsorption/desorption isotherms of NCM622 nanobricks, Figure S5: Typical XRD pattern and SEM images of NCM622 electrode after long-term 200 cycles at 0.5 C rate, Figure S6: Equivalent circuit model is used for fitting the experimental results. Rs: solution resistance, Rf: surface film resistance, related to Li-ions diffusion in the cathode electrolyte interface (CEI), and Rct: charge transfer resistance, CPE: constant phase element, Wo: Warburg element (open), Table S1: Unit cell parameters for the two fundamental phases of Ni(OH)_2_, Table S2: X-ray diffraction parameters of α-Ni(OH)_2_ based on JCPDS No.38-0715, Table S3: X-ray diffraction parameters of β-Ni(OH)_2_ based on JCPDS No. 14-0117, Table S4: The ICP-OES results of NCM622 nanobricks, Table S5: Atomic site information and crystallographic data for NCM622.

## References

[1] Armand M., Tarascon J.M. (2008). Building better batteries. Nature.

[2] Shen J., Xu X., Liu J., Liu Z., Li F., Hu R., Liu J., Hou X., Feng Y., Yu Y. (2019). Mechanistic Understanding of Metal Phosphide Host for Sulfur Cathode in High-Energy-Density Lithium-Sulfur Batteries. ACS Nano.

[3] Xu X., Liu J., Liu Z., Shen J., Hu R., Liu J., Ouyang L., Zhang L., Zhu M. (2017). Robust Pitaya-Structured Pyrite as High Energy Density Cathode for High-Rate Lithium Batteries. ACS Nano.

[4] Kim J., Lee H., Cha H., Yoon M., Park M., Cho J. (2018). Prospect and Reality of Ni-Rich Cathode for Commercialization. Adv. Energy Mater..

[5] Emani S., Liu C.H., Ashuri M., Sahni K., Wu J.P., Yang W.L., Nemeth K., Shaw L.L. (2019). Li_3_BN_2_ as a Transition Metal Free, High Capacity Cathode for Li-ion Batteries. Chemelectrochem.

[6] Lee W., Muhammad S., Sergey C., Lee H., Yoon J., Kang Y.-M., Yoon W.-S. (2020). Advances in the Cathode Materials for Lithium Rechargeable Batteries. Angew. Chem. Int. Ed..

[7] Li W., Erickson E.M., Manthiram A. (2020). High-nickel layered oxide cathodes for lithium-based automotive batteries. Nat. Energy.

[8] Liu W., Oh P., Liu X., Lee M.-J., Cho W., Chae S., Kim Y., Cho J. (2015). Nickel-Rich Layered Lithium Transition-Metal Oxide for High-Energy Lithium-Ion Batteries. Angew. Chem. Int. Ed..

[9] Zou L., Zhao W., Jia H., Zheng J., Li L., Abraham D.P., Chen G., Croy J.R., Zhang J.-G., Wang C. (2020). The Role of Secondary Particle Structures in Surface Phase Transitions of Ni-Rich Cathodes. Chem. Mater..

[10] Ronduda H., Zybert M., Szczesna-Chrzan A., Trzeciak T., Ostrowski A., Szymanski D., Wieczorek W., Rarog-Pilecka W., Marcinek M. (2020). On the Sensitivity of the Ni-rich Layered Cathode Materials for Li-ion Batteries to the Different Calcination Conditions. Nanomaterials.

[11] Kasnatscheew J., Evertz M., Streipert B., Wagner R., Klöpsch R., Vortmann B., Hahn H., Nowak S., Amereller M., Gentschev A.C. (2016). The truth about the 1st cycle Coulombic efficiency of LiNi_1/3_Co_1/3_Mn_1/3_O_2_ (NCM) cathodes. Phys. Chem. Chem. Phys..

[12] Ju X., Huang H., He W., Zheng H., Deng P., Li S., Qu B., Wang T. (2018). Surfactant-Assisted Synthesis of High Energy {010} Facets Beneficial to Li-Ion Transport Kinetics with Layered LiNi_0.6_Co_0.2_Mn_0.2_O_2_. ACS Sustain. Chem. Eng..

[13] Lin F., Markus I.M., Nordlund D., Weng T.-C., Asta M.D., Xin H.L., Doeff M.M. (2014). Surface reconstruction and chemical evolution of stoichiometric layered cathode materials for lithium-ion batteries. Nat. Commun..

[14] Flores E., Novák P., Aschauer U., Berg E. (2019). Cation Ordering and Redox Chemistry of Layered Ni-Rich Li_x_Ni_1-2y_Co_y_Mn_y_O_2_: An Operando Raman Spectroscopy Study. Chem. Mater..

[15] Sahni K., Ashuri M., He Q., Sahore R., Bloom I.D., Liu Y., Kaduk J.A., Shaw L.L. (2019). H_3_PO_4_ treatment to enhance the electrochemical properties of Li(Ni_1/3_Mn_1/3_Co_1/3_)O_2_ and Li(Ni_0.5_Mn_0.3_Co_0.2_)O_2_ cathodes. Electrochim. Acta.

[16] Xin F., Zhou H., Chen X., Zuba M., Chernova N., Zhou G., Whittingham M.S. (2019). Li-Nb-O Coating/Substitution Enhances the Electrochemical Performance of the LiNi_0.8_Mn_0.1_Co_0.1_O_2_ (NMC 811) Cathode. ACS Appl. Mater. Interfaces.

[17] Zheng J., Yang Z., Dai A., Tang L., Wei H., Li Y., He Z., Lu J. (2019). Boosting Cell Performance of LiNi_0.8_Co_0.15_Al_0.05_O_2_ via Surface Structure Design. Small.

[18] Wang X., Ding Y.-L., Deng Y.-P., Chen Z. (2020). Ni-Rich/Co-Poor Layered Cathode for Automotive Li-Ion Batteries: Promises and Challenges. Adv. Energy Mater..

[19] Shaw L., Ashuri M. (2019). Coating-A Potent Method to Enhance Electrochemical Performance of Li(Ni_x_Mn_y_Co_z_)O_2_ Cathodes for Li-ion Batteries. J. Adv. Mater. Lett..

[20] Haber S., Evenstein E., Saha A., Brontvein O., Kratish Y., Bravo-Zhivotovskii D., Apeloig Y., Leskes M., Noked M. (2020). Alkylated Li_x_Si_y_O_z_ Coating for Stabilization of Li-rich Layered Oxide Cathodes. Energy Storage Mater..

[21] Schipper F., Bouzaglo H., Dixit M., Erickson E.M., Weigel T., Talianker M., Grinblat J., Burstein L., Schmidt M., Lampert J. (2018). From Surface ZrO_2_ Coating to Bulk Zr Doping by High Temperature Annealing of Nickel-Rich Lithiated Oxides and Their Enhanced Electrochemical Performance in Lithium Ion Batteries. Adv. Energy Mater..

[22] Shevtsov A., Han H., Morozov A., Carozza J.C., Savina A.A., Shakhova I., Khasanova N.R., Antipov E.V., Dikarev E.V., Abakumov A.M. (2020). Protective Spinel Coating for Li_1.17_Ni_0.17_Mn_0.50_Co_0.17_O_2_ Cathode for Li-Ion Batteries through Single-Source Precursor Approach. Nanomaterials.

[23] Hall D.S., Gauthier R., Eldesoky A., Murray V.S., Dahn J.R. (2019). New Chemical Insights into the Beneficial Role of Al_2_O_3_ Cathode Coatings in Lithium-ion Cells. ACS Appl. Mater. Interfaces.

[24] Park K.-J., Jung H.-G., Kuo L.-Y., Kaghazchi P., Yoon C.S., Sun Y.-K. (2018). Improved Cycling Stability of Li[Ni_0.90_Co_0.05_Mn_0.05_]O_2_ Through Microstructure Modification by Boron Doping for Li-Ion Batteries. Adv. Energy Mater..

[25] Sun H.H., Ryu H.-H., Kim U.-H., Weeks J.A., Heller A., Sun Y.-K., Mullins C.B. (2020). Beyond Doping and Coating: Prospective Strategies for Stable High-Capacity Layered Ni-Rich Cathodes. ACS Energy Lett..

[26] Zhang C., Wan J., Li Y., Zheng S., Zhou K., Wang D., Wang D., Hong C., Gong Z., Yang Y. (2020). Restraining the polarization increase of Ni-rich and low-Co cathodes upon cycling by Al-doping. J. Mater. Chem. A.

[27] Sun Y.-K., Chen Z., Noh H.-J., Lee D.-J., Jung H.-G., Ren Y., Wang S., Yoon C.S., Myung S.-T., Amine K. (2012). Nanostructured high-energy cathode materials for advanced lithium batteries. Nat. Mater..

[28] Sun Y.-K., Myung S.-T., Kim M.-H., Prakash J., Amine K. (2005). Synthesis and Characterization of Li[(Ni_0.8_Co_0.1_Mn_0.1_)_0.8_(Ni_0.5_Mn_0.5_)_0.2_]O_2_ with the Microscale Core-Shell Structure as the Positive Electrode Material for Lithium Batteries. J. Am. Chem. Soc..

[29] Zeng X., Zhan C., Lu J., Amine K. (2018). Stabilization of a High-Capacity and High-Power Nickel-Based Cathode for Li-Ion Batteries. Chem.

[30] He L.-P., Li K., Zhang Y., Liu J. (2020). Structural and Electrochemical Properties of Low-Cobalt-Content LiNi_0.6+x_Co_0.2–x_Mn_0.2_O_2_ (0.0 ≤ x ≤ 0.1) Cathodes for Lithium-Ion Batteries. ACS Appl. Mater. Interfaces.

[31] Liu J., Kopold P., Wu C., van Aken P.A., Maier J., Yu Y. (2015). Uniform yolk-shell Sn_4_P_3_@C nanospheres as high-capacity and cycle-stable anode materials for sodium-ion batteries. Energy Environ. Sci..

[32] Liu J., Xu X., Shen J., Li F., Wang Z., Zhang D., Zuo S. (2020). Fe_3_O_4_@C Nanotubes Grown on Carbon Fabric as a Free-Standing Anode for High Performance Li-Ion Batteries. Chem. Eur. J..

[33] Chen Z., Chao D., Liu J., Copley M., Lin J., Shen Z., Kim G.-T., Passerini S. (2017). 1D nanobar-like LiNi_0.4_Co_0.2_Mn_0.4_O_2_ as a stable cathode material for lithium-ion batteries with superior long-term capacity retention and high rate capability. J. Mater. Chem. A.

[34] Kang H.J., Bari G., Lee T.G., Khan T.T., Park J.W., Hwang H.J., Cho S.Y., Jun Y.S. (2020). Microporous Carbon Nanoparticles for Lithium-Sulfur Batteries. Nanomaterials.

[35] Jiang B., Han C., Li B., He Y., Lin Z. (2016). In-Situ Crafting of ZnFe_2_O_4_ Nanoparticles Impregnated within Continuous Carbon Network as Advanced Anode Materials. ACS Nano.

[36] Jiang B., He Y., Li B., Zhao S., Wang S., He Y.B., Lin Z. (2017). Polymer-Templated Formation of Polydopamine-Coated SnO_2_ Nanocrystals: Anodes for Cyclable Lithium-Ion Batteries. Angew. Chem. Int. Ed..

[37] Liu Y., Huang L., Ding Z., Wang J., Wu J., Zhang H., Lavorgna M. (2019). On the tailoring the 1D rod-like hierarchical nano/micro LiNi_0.8_Co_0.15_Al_0.05_O_2_ structure with exposed (101) plane by template method. J. Alloys Compd..

[38] Xiang W., Liu W.-Y., Zhang J., Wang S., Zhang T.-T., Yin K., Peng X., Jiang Y.-C., Liu K.-H., Guo X.-D. (2019). Controlled synthesis of nickel-rich layered oxide cathodes with preferentially exposed {010} active facets for high rate and long cycling stable lithium-ion batteries. J. Alloys Compd..

[39] Zhang L., Li N., Wu B., Xu H., Wang L., Yang X.-Q., Wu F. (2015). Sphere-Shaped Hierarchical Cathode with Enhanced Growth of Nanocrystal Planes for High-Rate and Cycling-Stable Li-Ion Batteries. Nano Lett..

[40] Chen M., Jin X., Chen Z., Zhong Y., Liao Y., Qiu Y., Cao G., Li W. (2019). A cross-like hierarchical porous lithium-rich layered oxide with (110)-oriented crystal planes as a high energy density cathode for lithium ion batteries. J. Mater. Chem. A.

[41] Jiang M., Zhang Q., Wu X., Chen Z., Danilov D.L., Eichel R.-A., Notten P.H.L. (2020). Synthesis of Ni-Rich Layered-Oxide Nanomaterials with Enhanced Li-Ion Diffusion Pathways as High-Rate Cathodes for Li-Ion Batteries. ACS Appl. Energy Mater..

[42] Hall D.S., Lockwood D.J., Bock C., MacDougall B.R. (2015). Nickel hydroxides and related materials: A review of their structures, synthesis and properties. Proc. Math. Phys. Eng. Sci..

[43] Liu A., Dahn J. (2019). The Formation of Layered Double Hydroxide Phases in the Coprecipitation Syntheses of [Ni_0.80_Co_0.15_]_(1__-x)/0.95_Al_x_(OH)_2_(anion^n^^–^)_x/n_ (x = 0–0.2, n = 1, 2). ChemEngineering.

[44] Song Y., Wang M., Li J., Liu Y., Cui H. (2020). Nanosheets self-supported structure in the orderly porous spheres of Co/Mn ions co-substituted α-Ni(OH)_2_ for high-performance supercapacitors. J. Sol-Gel Sci. Technol..

[45] Wei W., Ye W., Wang J., Huang C., Xiong J.B., Qiao H., Cui S., Chen W., Mi L., Yan P. (2019). Hydrangea-like alpha-Ni_1/3_Co_2/3_(OH)_2_ Reinforced by Ethyl Carbamate “Rivet” for All-Solid-State Supercapacitors with Outstanding Comprehensive Performance. ACS Appl. Mater. Interfaces.

[46] Duquesne E., Betelu S., Seron A., Ignatiadis I., Perrot H., Debiemme-Chouvy C. (2020). Tuning Redox State and Ionic Transfers of Mg/Fe-Layered Double Hydroxide Nanosheets by Electrochemical and Electrogravimetric Methods. Nanomaterials.

[47] Gao M., Sheng W., Zhuang Z., Fang Q., Gu S., Jiang J., Yan Y. (2014). Efficient water oxidation using nanostructured alpha-nickel-hydroxide as an electrocatalyst. J. Am. Chem. Soc..

[48] Zhang X.-D., Shi J.-L., Liang J.-Y., Yin Y.-X., Zhang J.-N., Yu X.-Q., Guo Y.-G. (2018). Suppressing Surface Lattice Oxygen Release of Li-Rich Cathode Materials via Heterostructured Spinel Li_4_Mn_5_O_12_ Coating. Adv. Mater..

[49] Meng F., Hu R., Chen Z., Tan L., Lan X., Yuan B. (2021). Plasma assisted synthesis of LiNi_0.6_Co_0.2_Mn_0.2_O_2_ cathode materials with good cyclic stability at subzero temperatures. J. Energy Chem..

[50] Mo Y., Guo L., Cao B., Wang Y., Zhang L., Jia X., Chen Y. (2019). Correlating structural changes of the improved cyclability upon Nd-substitution in LiNi_0.5_Co_0.2_Mn_0.3_O_2_ cathode materials. Energy Storage Mater..

[51] Li Y., Bai Y., Wu C., Qian J., Chen G., Liu L., Wang H., Zhou X., Wu F. (2016). Three-dimensional fusiform hierarchical micro/nano Li_1.2_Ni_0.2_Mn_0.6_O_2_ with a preferred orientation (110) plane as a high energy cathode material for lithium-ion batteries. J. Mater. Chem. A.

[52] Xu M., Fei L., Zhang W., Li T., Lu W., Zhang N., Lai Y., Zhang Z., Fang J., Zhang K. (2017). Tailoring Anisotropic Li-Ion Transport Tunnels on Orthogonally Arranged Li-Rich Layered Oxide Nanoplates toward High-Performance Li-Ion Batteries. Nano Lett..

[53] Li J., Yao R., Cao C. (2014). LiNi_1/3_Co_1/3_Mn_1/3_O_2_ Nanoplates with {010} Active Planes Exposing Prepared in Polyol Medium as a High-Performance Cathode for Li-Ion Battery. ACS Appl. Mater. Interfaces.

[54] Zhang X., Mauger A., Lu Q., Groult H., Perrigaud L., Gendron F., Julien C.M. (2010). Synthesis and characterization of LiNi_1/3_Mn_1/3_Co_1/3_O_2_ by wet-chemical method. Electrochim. Acta.

[55] Shen Z., Cao L., Rahn C.D., Wang C.Y. (2013). Least Squares Galvanostatic Intermittent Titration Technique (LS-GITT) for Accurate Solid Phase Diffusivity Measurement. J. Electrochem. Soc..

[56] Märker K., Reeves P.J., Xu C., Griffith K.J., Grey C.P. (2019). Evolution of Structure and Lithium Dynamics in LiNi_0.8_Mn_0.1_Co_0.1_O_2_ (NMC811) Cathodes during Electrochemical Cycling. Chem. Mater..

[57] Funabiki A., Inaba M., Ogumi Z. (1997). Ac impedance analysis of electrochemical lithium intercalation into highly oriented pyrolytic graphite. J. Power Sources.

