# Supplementary material for: Ni-Rich Layered Oxide with Preferred Orientation (110) Plane as a Stable Cathode Material for High-Energy Lithium-Ion Batteries
Source: Nanomaterials (Basel). 2020 Dec 11;10(12):2495. doi: 10.3390/nano10122495 (PMC7764293; doi:10.3390/nano10122495)
Supplement: Supplementary file 1 [file nanomaterials-10-02495-s001.pdf]

nanomaterials

Supplementary Materials

Ni-Rich Layered Oxide with Preferred Orientation (110) Plane as a Stable Cathode Material for High-Energy Lithium-Ion Batteries

Fangkun Li, Zhengbo Liu, Jiadong Shen, Xijun Xu, Liyan Zeng, Yu Li, Dechao Zhang, Shiyong Zuo and Jun Liu *

Guangdong Provincial Key Laboratory of Advanced Energy Storage Materials, School of Materials Science and Engineering, South China University of Technology, Guangzhou 510641, China; mslifk@scut.edu.cn (F.L.); 201810103813@mail.scut.edu.cn (Z.L.); 201910103734@mail.scut.edu.cn (J.S.); xuxijun2019@scut.edu.cn (X.X.); 201820117753@mail.scut.edu.cn (L.Z.); 201820117766@mail.scut.edu.cn (Y.L.); 201810103808@mail.scut.edu.cn (D.Z.); shyzuo@scut.edu.cn (S.Z.)

* Correspondence: msjliu@scut.edu.cn

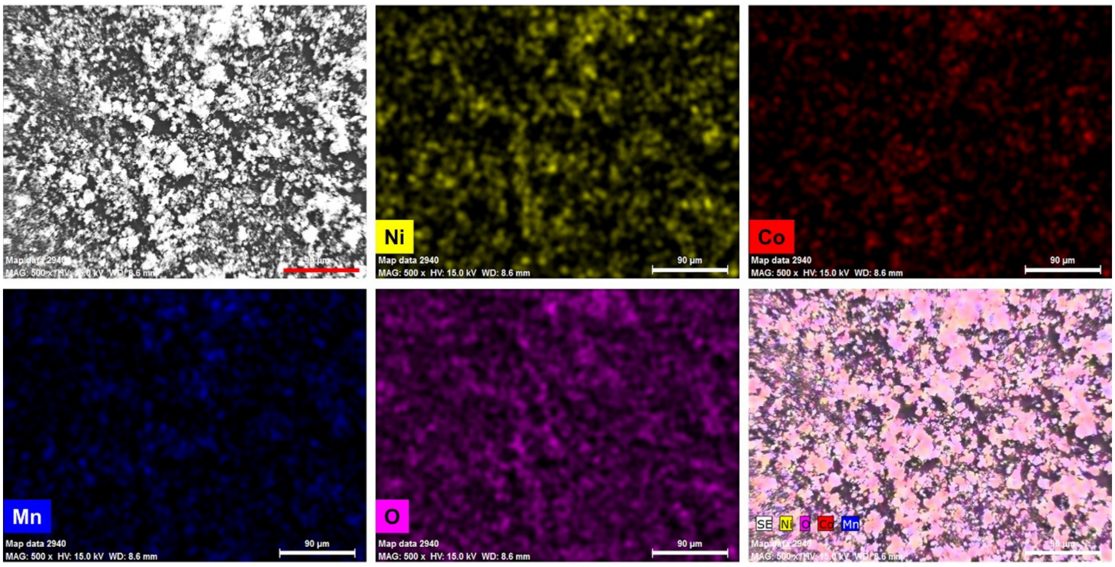

Figure S1. SEM image and elemental EDS mapping of Ni, Co, Mn, O and all elements for the intermediate oxides composite, the scale bar is 90 µm in all figures.

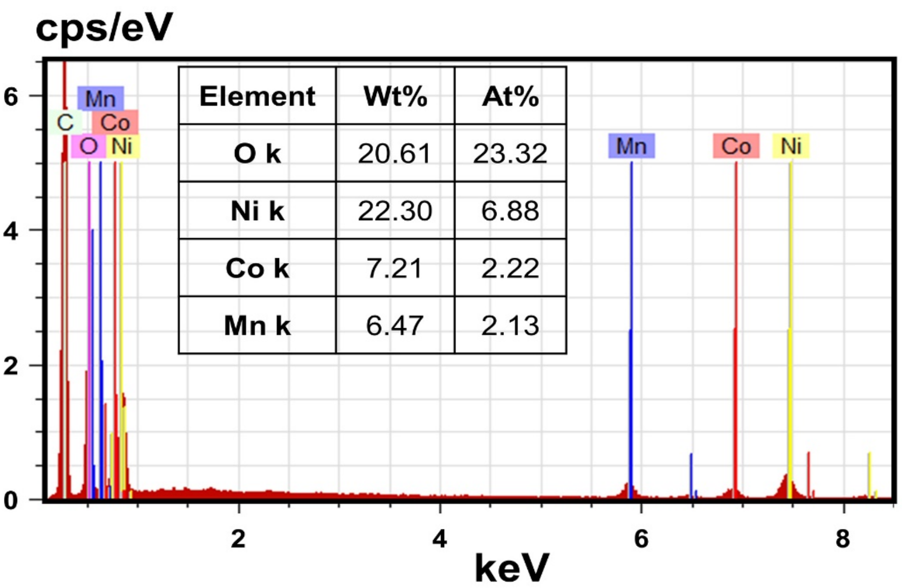

Figure S2. EDS spectrum and corresponding element composition of intermediate oxides composite.

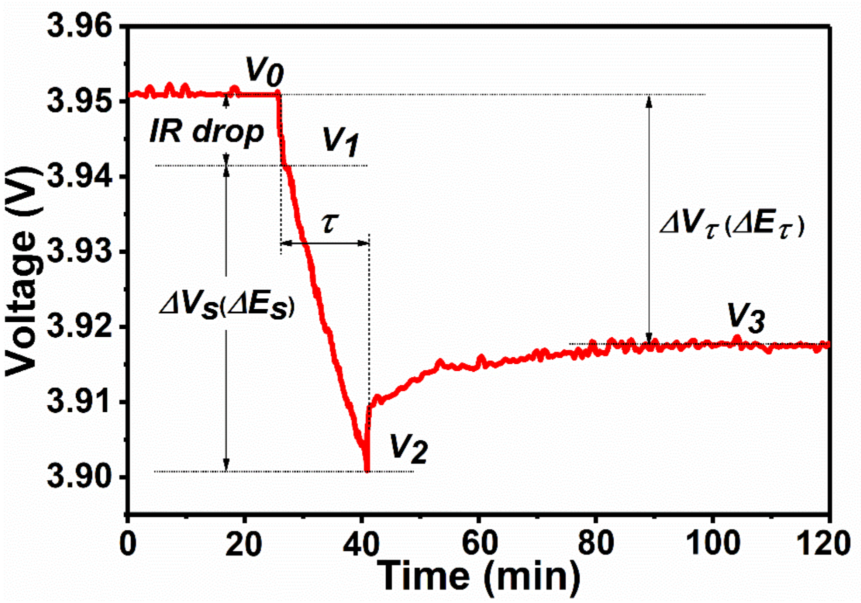

Figure S3. Applied current plus vs. cell voltage for a single titration step of GITT curves.

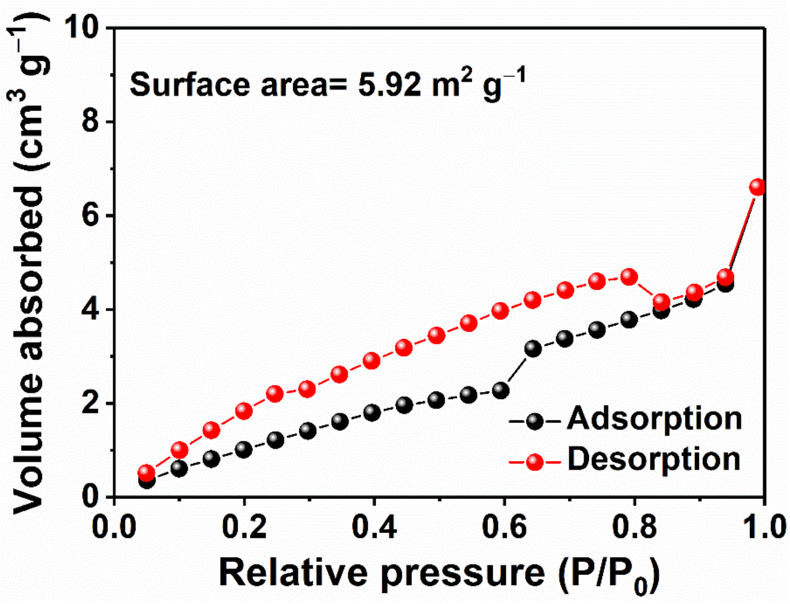

Figure S4. N_2 adsorption/desorption isotherms of NCM622 nanobricks.

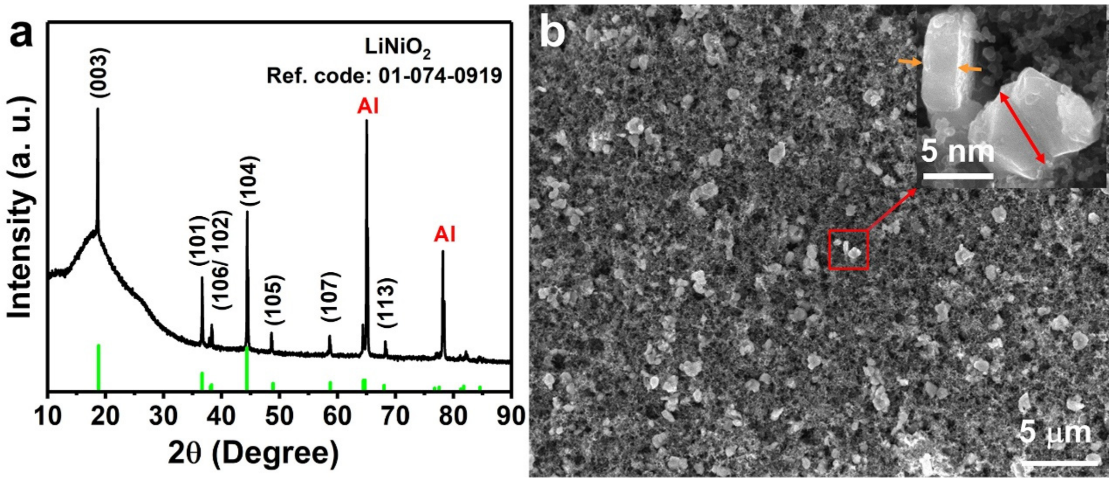

Figure S5. Typical XRD pattern and SEM images of NCM622 electrode after long-term 200 cycles at 0.5 C rate.

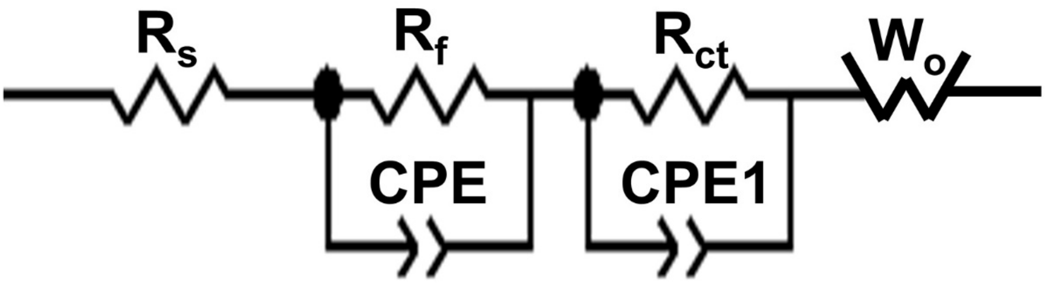

Figure S6. Equivalent circuit model is used for fitting the experimental results. R_s: solution resistance, R_f: surface film resistance, related to Li-ions diffusion in the cathode electrolyte interface (CEI), and R_{ct}: charge transfer resistance, *CPE*: constant phase element, W_o: Warburg element (open).

Table S1. Unit cell parameters for the two fundamental phases of $Ni(OH)_2$.

.	**α-$Ni(OH)_2$**	**β-$Ni(OH)_2$**
Space group	$D_{3d}^{1}/P\bar{3}Im$/No.162	$D_{3d}^{1}/P\bar{3}Im$/No.164
a = b	3.08 Å	3.126 Å
c	8.0 Å	4.605 Å
	$\alpha = \beta = 90°, \gamma = 120°$	

Table S2. X-ray diffraction parameters of α-$Ni(OH)_2$ based on JCPDS No.38-0715.

**Miller indices (hkl)**	**d (Å)**	**2θ (°)**	**I (a. u.)**
(003)	7.79	11.349	100.0
(006)	3.91	22.735	70.0
(101)	2.68	33.458	50.0
(012)	2.60	34.412	50.0
(015)	2.32	38.77	50.0
(018)	1.97	45.99	20.0
(110)	1.54	59.98	50.0
(113)	1.51	61.25	20.0

Table S3. X-ray diffraction parameters of β-$Ni(OH)_2$ based on JCPDS No. 14-0117.

**Miller indices (hkl)**	**d (Å)**	**2θ (°)**	**I (a. u.)**
(001)	4.61	19.258	100.0
(100)	2.71	33.064	45.0
(101)	2.33	38.541	100.0
(002)	2.30	39.098	2.2
(102)	1.75	52.100	35.0
(110)	1.56	59.052	25.0
(003)	1.53	60.240	<1
(111)	1.48	62.73	16.0

Table S4. The ICP-OES results of NCM622 nanobricks.

	**Li**	**Ni**	**Co**	**Mn**
Weight ratio (%)	6.97	35.03	11.50	11.12
Molar ratio (%)	1.03	0.61	0.20	0.20

Table S5. Atomic site information and crystallographic data for NCM622.

**Atom**	**Wyck.**	**a**	**b**	**c**	**Occ.**	**Ui/Ue*100**
Li1	3a	0	0	0	0.946	1.89
Ni2	3a	0	0	0	0.054	1.89
Li2	3b	0	0	0.5	0.010	0.70
Ni1	3b	0	0	0.5	0.590	0.70
Co1	3b	0	0	0.5	0.200	0.70
Mn1	3b	0	0	0.5	0.200	0.70
O2	6c	0	0	0.25661(12)	1.000	0.54
Lattice parameters (Å)			a = b = 2.86770(4), c = 14.21426(27), α = β = 90°, γ = 120°			
Cell volume			101.2333(28) $Å^3$			
